# Curcumin ameliorates chronic *Toxoplasma gondii* infection-induced affective disorders through modulation of proinflammatory cytokines and oxidative stress 

**DOI:** 10.22038/IJBMS.2023.68487.14937

**Published:** 2023-04

**Authors:** Fatemeh Moradi, Nasrin Dashti, Amirali Farahvash, Farinaz Baghaei Naeini, Mitra Zarebavani

**Affiliations:** 1 School of Medicine, Mazandaran University of Medical Sciences, Sari, Iran; 2 Department of Clinical Laboratory Sciences, School of Allied Medical Sciences, Tehran University of Medical Sciences, Tehran, Iran; 3 Özel Medipark Tıp Merkezi, Ankara, Turkey; 4 Science and Research branch, Islamic Azad University, Tehran, Iran

**Keywords:** Anxiety, Curcumin, Depression, Neuroinflammation, Oxidative stress, Toxoplasma

## Abstract

**Objective(s):**

Long-term infection with *Toxoplasma gondii *is associated with affective disorders (i.e., anxiety and depression) in adults. We aimed to explore the effects of curcumin (CR) on anxiety- and depressive-like behaviors in mice infected with *T. gondii*.

**Materials and Methods:**

Animals were studied in five groups: Control, Model, Model + CR20, 40, and 80 (with IP injection of 20, 40, and 80 mg/kg CR). *T. gondii* infection was prolonged for four weeks. The animals were then treated with CR or vehicle for two weeks and evaluated by behavioral tests at the end of the study. Hippocampal levels of oxidative stress biomarkers (superoxide dismutase; SOD, glutathione; GSH, and malondialdehyde; MDA) and gene expression and protein levels of hippocampal proinflammatory mediators (interleukin-1β; IL-1β, IL-6, IL-18, and tumor necrosis factor- α; TNF-α) were determined.

**Results:**

Behavioral tests confirmed that long-term infection with *T. gondii* led to anxiety- and depressive-like behaviors. Antidepressant effects of CR were linked to modulation of oxidative stress and cytokine network in the hippocampal region of infected mice. These results showed that CR reduced anxiety and depression symptoms via regulation of oxidative stress and proinflammatory cytokines in the hippocampus of *T. gondii*-infected mice.

**Conclusion:**

Therefore, CR can be used as a potential antidepressant agent against T. gondii-induced affective disorders.

## Introduction

*Toxoplasma gondii* (*T. gondii*) is an intracellular apicomplexan protozoan parasite that infects humans and causes the toxoplasmosis disease. One-third of people worldwide are infected with *T. gondii* (1, 2). Members of the cat family serve as the definitive host for the sexual stages of *T. gondii* (3). Although *T. gondii* does not lead to serious complications in most adult humans, infecting pregnant women with this parasite for the first time can lead to serious illness (4). Several studies have reported that acute and latent toxoplasmosis is associated with mental disorders (including anxiety, depression, bipolar disorder, and schizophrenia) (5) and cognitive dysfunction (6). *T. gondii* can invade neurons (neurotropism) and impair brain function with lifelong persistence in the host brain (3). According to histopathological studies, *T. gondii* infection may be related to structural changes in the brain (7). The presence of* T. gondii* cysts in neurons can alter the release of neurotransmitters, e.g., glutamate, dopamine, serotonin, and gamma-aminobutyric acid (8, 9). Oxidative stress (OS) has been implicated in the pathogenicity of *T. gondii*-induced brain tissue damage. A significant decrease was observed in the levels of anti-oxidant enzymes in animals infected with *T. gondii* (10). Neuroinflammation plays an important role in onset of neuropsychological disorders. While direct infection of the central nervous system (CNS) with *T. gondii* has been implicated in the risk of mental and behavioral disorders, systemic inflammation following chronic infections indirectly contributes to the induction of these conditions (11, 12). Furthermore, chronic *T. gondii* infection has been reported to induce anxiety and depressive behavior associated with up-regulation of inflammatory cytokines in the central nervous system (13).

The therapeutic approaches of *T. gondii* are still limited, mainly for the treatment of the chronic phase of the infection (14). The combination of sulfadiazine, pyrimethamine, and folinic acid is the most effective therapeutic program, able to suppress the proliferation of *T. gondii* tachyzoites by blocking the folate synthesis pathways (15, 16). However, this approach is not effective enough to inhibit latent tissue cysts (bradyzoites) and exerts critical adverse effects (17). Curcumin (CR) is an active natural compound isolated from the dietary spice turmeric (*Curcuma longa *Linn) and exhibits various pharmacological effects, including anti-oxidant, anti-inflammatory, and antitumor (18). Due to the safety and effectiveness of CR, it can be used for a variety of diseases and conditions (19). Additionally, its activity against *T. gondii*-induced infections in mice has been demonstrated (20). The antidepressant effects of this component are linked to multiple mechanisms of action (21). 

To the best of the authors’ knowledge, there are no reports on evaluating the effects of CR on chronic toxoplasmosis-induced affective disorders. In this study, we aimed to investigate the potential effects of CR against *T. gondii-*related anxiety- and depressive-like behaviors by estimating the levels of OS and inflammatory biomarkers in the hippocampus of the infected mice. 

## Materials and Methods

***Study design and animals***

**Figure 1:**
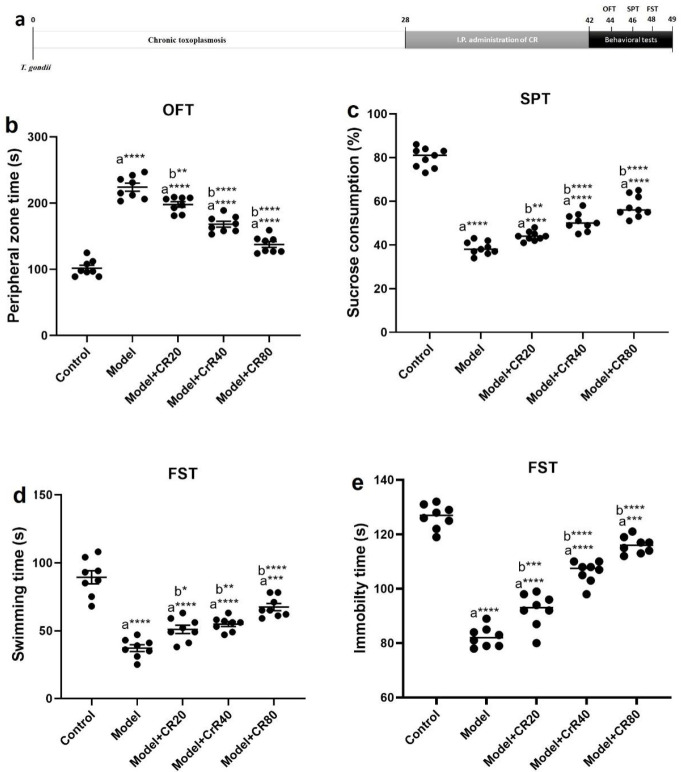
Effect of CR on behavioral alterations in chronic *Toxoplasma gondii *infection-induced anxiety and depression model of mice. a) Schematic overview of experimental design, b) Time spent in the peripheral zone of the arena (OFT), c) Sucrose consumption percentage (SPT), d) Swimming time (FST), and e) Immobility time (FST). Values: mean ± SEM (n = 8). a: vs control group, b: vs model group, *P<0.05, **P<0.01, ***P<0.001, and ****P<0.0001

The experiment was accepted by the Animal Ethics Community of Tehran University of Medical Sciences (TUMS), Tehran, Iran and conducted based on the “Guide to the Care and Use of Laboratory Animals, 8th Edition”. A total of 40 male BALB/c mice male mice with an average weight of 12–16 g (3-4 weeks) were provided by the TUMS animal laboratory and maintained under a noise, temperature, and light-controlled (12 hr light/dark cycle) condition in polypropylene cages. Mice were provided food and water *ad libitum*. After adaptation, animals were studied in five groups (n = 8 per group): Control: uninfected animals received normal saline for two weeks; Model: *T. gondii* -induced mice treated with normal saline (vehicle) for two weeks; Model + CR: *T. gondii*-induced mice treated with CR (20, 40, or 80 mg/kg/intraperitoneal) for two weeks. The procedure is summarized in Figure 1a.

***Parasite preparation and***

*T. gondii* tachyzoites (RH strain) were collected every two days by serial passages in the peritoneal cavity of BALB/c mice. As previously described, the brain tissue of mice infected with *T. gondii* tissue cysts was isolated and suspended in phosphate buffer saline (PBS), at pH 7.2, and then filtered through gauze. Subsequently, 0.1 ml of brain suspension containing 100 tissue cysts was injected intraperitoneally into each male mouse (22). Anti *T. gondii* IgG antibody (Toxoscreen DA, Biomérieux, Lyon, France) was used to confirm toxoplasmosis in each mouse by the modified agglutination test. 

***Neurobehavioral assessment***

Finally, eight animals in each group were used for the neurobehavioral tests, including forced swimming test (FST), open field test (OFT), and sucrose preference test (SPT). 

*Open field test*

OFT is usually performed to investigate the anxiety behavior of animals (23). The apparatus was an open field (50 × 50 × 40 cm^3^) consisting of a clear Plexiglas box with a floor divided into 16 equal squares. A single mouse was placed on the center of the floor, and its behaviors were videotaped for 5 min to analyze the time spent in the peripheral zone (24).

*Sucrose preference test*

This test was performed based on a previously described protocol for assessing anhedonia (25). Mice were adapted to a sucrose solution (1% sucrose [w/v]) by placing two bottles in each cage for 24 hr. Then, each mouse was placed individually in a cage, and a bottle was replaced with a bottle containing water (volume: 100 ml). After one hour, the volume of solution consumed was determined and the sucrose consumption (%) was calculated according to the following formula:

Sucrose consuption %=Sucrose consuption (ml)Sucrose consuption ml+Water consuption (ml)×100

*Forced swimming test*

FST is a well-known paradigm for the assessment of depressive phenotypes in animal models (26). Briefly, a polyvinyl chloride cylinder (20 cm in diameter, 30 cm in height, and 2 L in volume) filled with fresh water to a depth of 20 cm (at a temperature of 24 ± 2 °C water) was used. Each mouse was placed in the cylinder, and the camera recorded the condition of each animal for 6 min. Immobility and swimming time were calculated for the last 4 min (27).

***Tissue preparation***

At the end of the experiment, the animals were sacrificed and the brain tissue was quickly dissected. The hippocampus was then isolated and stored in a refrigerator at −80 °C pending molecular studies. Hippocampal tissues were homogenized for evaluation of three main factors (n=4): 1) determination of OS biomarkers including malondialdehyde (MDA), superoxide dismutase (SOD), and glutathione (GSH); 2) Gene expression analysis of cytokines, including tumor necrosis factor α (TNFα), interleukin-1β (IL-1β), IL6, and IL-18 real-time quantitative PCR (qPCR) (n = 4); and 3) Determination of protein levels of the mentioned cytokines using the ELISA technique (n = 4). 

***Determination of anti-oxidant biomarkers***

The homogenized tissue (10% w/v in PBS) was centrifuged to collect the supernatants. Subsequently, the hippocampal levels of OS biomarkers were measured using MDA, GSH, and SOD assay kits (ZellBio GmbH, Ulm, Germany) in accordance with the manufacturer’s instructions.

***qPCR***

Total RNA was extracted from the hippocampus with Trizol reagent. The purity was evaluated with a spectrophotometer (Quawell, USA). PrimeScript reverse transcription kit (TAKARA Bio, Japan) was used for cDNA synthesis. According to the manufacturer’s instructions, 1 μg of RNA was used to determine reverse transcription. qPCR was done using the SYBR-Green kit (Takara Bio Inc.) and a Cycler (Light Cycler 2.0, Roche) according to the manufacturer’s protocols. Relative expression levels were measured by the formula 2−ΔΔCt. The mRNA levels of IL-1β, IL-6, IL-18, and TNFα were determined and standardized using glyceraldehyde 3-phosphate dehydrogenase (GAPDH) as the housekeeping gene. Data were presented as fold changes in target gene expression normalized to GAPDH. The primers used in the experiments are shown in Table 1.

***ELISA***

The levels of inflammatory cytokines (TNFα, IL-1β, IL-6, and IL-18) were assayed using ELISA kits (Abcam) according to the manufacturer’s instructions. A microplate reader (Varioskan Flash, Thermo Scientific, USA) was used for the measurements (450 nm). 

***Statistics***

The mean ± SEM was used to express the data. GraphPad Prism software (version 6.0) was used for data analysis. Statistical analysis was done by one-way ANOVA with Tukey’s *post hoc* test. *P<*0.05 was statistically significant. 

CR: Curcumin

**Table 1:** Primer sequences for qPCR in the study

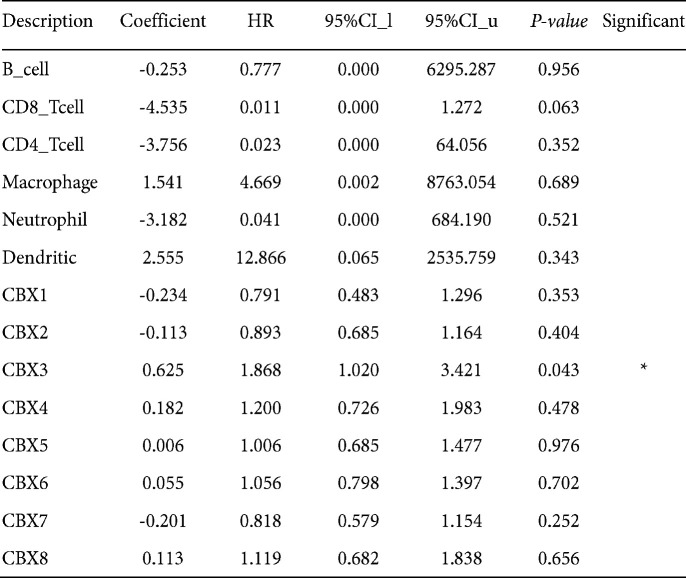

**Figure 2:**
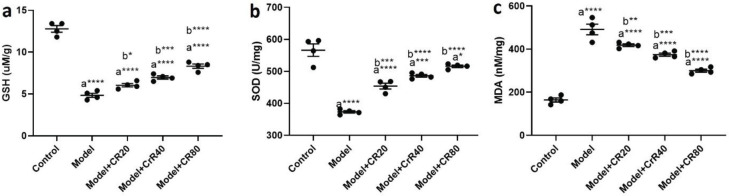
Effect of CR on the hippocampal levels of oxidative stress markers of chronic *Toxoplasma gondii* infection-induced depression mice model. a) GSH, b) SOD, and c) MDA. Values: mean ± SEM (n = 4). a: vs control group, b: vs model group, *P<0.05, *** *P*<0.001, and ****P<0.0001

CR: Curcumin; GSH: Glutathione; SOD: Superoxide dismutase; MDA: Malondialdehyde

**Figure 3:**
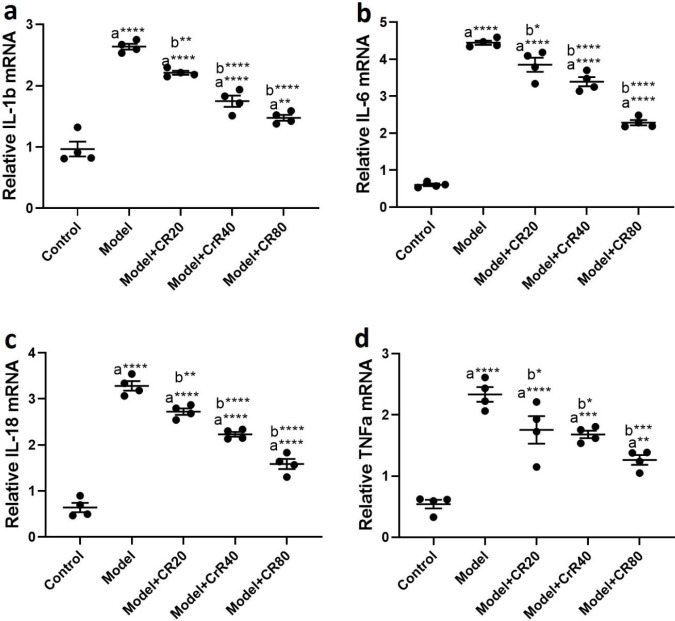
Effect of CR on the mRNA levels of hippocampal proinflammatory cytokines in mice infected by *Toxoplasma gondii*. a) IL-1β, b) IL-6, c) IL-18, and d) TNF-α. Values: Mean ± SEM (n = 4). a: vs control group, b: vs model group, *P<0.05, **P<0.01, ***P<0.001, and ****P<0.0001

CR: Curcumin

**Figure 4:**
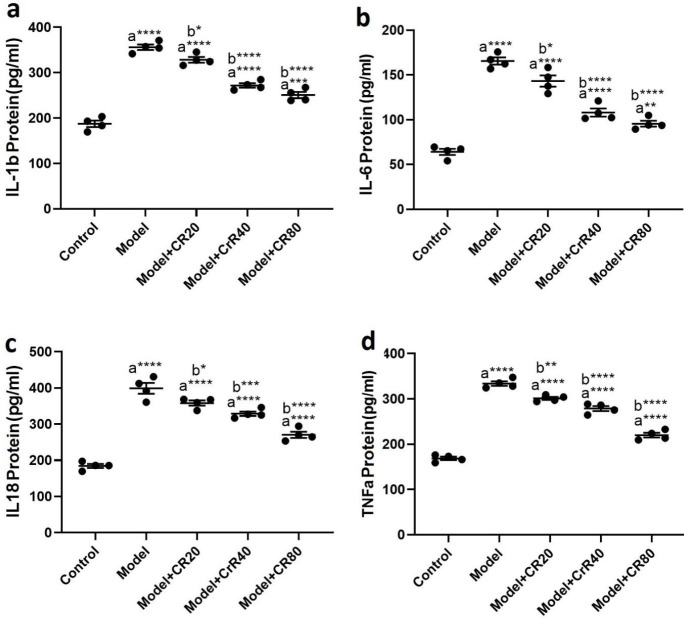
Effect of CR on the hippocampal levels of proinflammatory cytokines. in mice infected by *Toxoplasma gondii.* a) IL-1β, b) IL-6, c) IL-18, and d) TNF-α. Values: mean ± SEM (n = 4). a: vs control group, b: vs model group, *P<0.05, **P<0.01, ***P<0.001, and ****P<0.0001

CR: Curcumin

## Results

***Behavioral studies***

According to the OFT results (Figure 1b), the mean time spent in the peripheral zone of the model group was longer than that of the control group (*P<*0.0001). Treatment with CR (20, 40, and 80 mg/kg) significantly reduced the time spent in the peripheral zone compared with the model group (*P<*0.05). 

According to Figure 1c, SPT results showed that the percentage of sucrose consumption by depressed animals was significantly reduced compared with uninfected animals (*P<*0.0001). CR treatment dose-dependently improved the percentage of sucrose consumption in the model + CR20, model + CR40, and model + CR80 groups compared with the model group (*P<*0.05). 

According to the FST results (Figure 1d), infection of the animals with *T. gondii* significantly reduced the swimming time (*P<*0.05). Treatment with CR significantly reversed the effects of long-term *T. gondii* infection in the model + CR20, model + CR40, and model + CR80 groups compared with the model group (*P<*0.05). Moreover, the immobility time (Figure 1e) was significantly prolonged in the infected group (*P<*0.05). Administration of CR significantly reduced the immobility time in the model + CR20, model + CR40, and model + CR80 groups compared with the model group (*P<*0.05).

***Hippocampal levels of oxidative stress markers***

According to Figure 2, protein levels of OS biomarkers (GSH, SOD, and MDA) were determined in the hippocampus of infected animals*. *The hippocampal activity of GSH (*P<*0.0001, Figure 2a) and SOD (*P<*0.0001, Figure 2b) were significantly reduced in the infected group compared with the control group. Moreover, hippocampal levels of MDA (*P<*0.0001, Figure 2c) were significantly increased in the infected group compared with the control group. Administration of CR dose-dependently modulated hippocampal levels of OS biomarkers in infected animals. 

***mRNA levels of hippocampal proinflammatory cytokines***

*T. gondii* infection significantly increased the gene expression of proinflammatory cytokines, including TNFα (*P<*0.0001, Figure 3a), IL6 (*P<*0.0001, Figure 3b), IL-1β (*P<*0.0001, Figure 3c), and IL-18 (*P<*0.0001, Figure 3d) in the hippocampus of infected animals compared with the control group. The qPCR results demonstrated that CR treatment attenuated the elevated levels of proinflammatory cytokines in the hippocampus after *T. gondii* infection. 

***Effects of CR on protein levels of proinflammatory cytokines***

The ELISA results revealed that *T. gondii* significantly increased the protein levels of proinflammatory cytokines, including TNFα (*P<*0.0001, Figure 4a), IL6 (*P<*0.0001, Figure 4b), IL-1β (*P<*0.0001, Figure 4c), and IL-18 (*P<*0.0001, Figure 4d) in the hippocampus of the infected animals compared with the control group. CR treatment reduced elevated levels of proinflammatory cytokines in the hippocampus in infected mice.

## Discussion

In this study, we used CR (20, 40, and 80 g/kg) to treat chronic *T. gondii *infection-induced affective disorders. According to the results of behavioral tests, a four-week exposure of animals to chronic *T. gondii* infection led to anxiety- and depressive-like behaviors in mice. In this study, we used OPT, SPT, and FST to examine animal behavior. The OFT results showed that the time spent in the peripheral zone was prolonged in the infected group. Additionally, FST results showed an increase in the immobility time and a decrease in the swimming time in infected animals. The percentage of sucrose consumption decreased in the model group. All of these findings confirmed that anxiety- and depressive-like behavior occurs in infected animals. *T. gondii*, an intracellular protozoan parasite, can infect humans and other warm-blooded vertebrates, such as livestock and cats. In recent years, several studies have confirmed that long-term infection of the CNS with *T. gondii* may be associated with neuropsychiatric disorders, including dementia, schizophrenia, and personality changes (28-32). *T. gondii* has emerged as an attractive candidate for a possible cause of mood alterations in infected patients (32). 

Our results indicated that *T. gondii* infection affected brain function through dysregulation of OS biomarkers (SOD, GSH, and MDA) and proinflammatory cytokines (TNFα, IL-6, IL-1β, and IL-18) in the hippocampus. OS has been implicated as a common factor in the pathogenesis of anxiety and depression (33, 34). The presence of OS in the CNS as a result of increased levels of reactive oxygen species (ROS) can activate the NLRP3 inflammasome in microglial cells. The NLRP3 inflammasome, an intracellular complex, is an important mediator of cytokine production (35). At the same time, activated microglia can produce high levels of pro-inflammatory cytokines, which in turn worsens OS (36). Neuroinflammation plays a critical role in the pathogenesis of neuropsychiatric disorders, including depression and bipolar disorder (37-39). Chronic neuroinflammation is linked to the disruption of neural plasticity and neurogenesis in the hippocampus, leading to anxiety and mood disorders (40). 

Microglia, primary immune cells of the brain, and astrocytes appear to be critical components of the innate immune system in the CNS (41, 42). Activation of microglia in response to external stimuli, such as infections, traumatic brain injury, autoimmune products, and toxic agents, triggers neuroinflammation (39). Furthermore, *T. gondii* infection leads to persistent neuroinflammation (39), which leads to neuropsychological deficits (43, 44) and behavioral alterations (45, 46). An increase in the level of interferon-γ (IFN-γ) to control *T. gondii* growth causes tryptophan depletion and activation of indoleamine-2,3-dioxygenase, resulting in reduced CNS levels of serotonin and induction of depressive symptoms (47). *T. gondii* infection was shown to promote neuroinflammation through activation of cytokine networks (up-regulation of TNFα, IL-6, and IL-1β) in mice (48). Although TNF-α, IL-6, and IL-1β play crucial roles as acute phase proteins, they may act differently in the pathology of depression. Microglia in the brain and macrophages in the blood are responsible for the production of proinflammatory cytokines (49). IL-1β is essential in the pathogenesis and pathophysiology of anxiety- and depressive-like behavior (50, 51). IL-1β stimulation leads to several depressive-like behaviors in animal models, and suppression of IL-1β overexpression has shown a beneficial antidepressant-like effect (52).

Dietary supplementations have been revealed to inhibit neuroinflammatory signals and reverse inflammation-associated abnormalities in the hippocampus (53). In the present study, animals were treated with CR (20, 40, 80 mg/kg) for two weeks. Our findings confirmed that CR administration improved anxiety- and depressive-like behaviors by reducing time spent in the peripheral zone in OFT, increasing swimming time and reducing immobility time in FST, and increasing sucrose consumption in SPT. Moreover, this component could successfully enhance anti-oxidant defense via increasing GSH and SOD. In addition, CR could decrease lipid peroxidation by reducing MDA levels in the hippocampus. Elevated hippocampal proinflammatory cytokines after* T. gondii *infection were also reversed by CR administration. According to the literature, CR exerts a broad spectrum of pharmacological and biological characteristics, including anti-oxidant, anti-inflammatory, and antiviral (54). Moreover, the parasiticidal and cytotoxic properties of CR have been reported in helminthic parasites, e.g., *Schistosoma mansoni* (55) and *Schistosoma japonicum* (56), and a variety of protozoan parasites, e.g., *Giardia lamblia* (57), *Leishmania* (58), *Plasmodium falciparum* (59), and *Trypanosoma cruzi* (60). 

Recently, the efficiency of CR against *T. gondii *infection has been examined. Its inhibitory activity against the spread of parasites makes it a potential therapeutic agent against toxoplasmosis (61). Several studies have indicated that herbal medicines or their derivatives have potential anxiolytic and antidepressant effects (62, 63). A growing body of evidence has confirmed the anxiolytic and antidepressant effects of CR through various mechanisms. In an animal study, administration of CR (50 mg/kg) improved FST and tail suspension test (TST) scores by suppressing monoamine oxidase enzymes and regulating concentrations of the neurotransmitters dopamine and serotonin in the brain (64). The anti-inflammatory effects of CR are related to the inhibition of cytokine production. CR could prevent the production of mature IL-1β and inhibit insulin resistance as a consequence of a high-fat diet in a mouse model (65). In stressed animals, CR could attenuate depressive behaviors via modulation of NF-κB/NLRP3 signaling and subsequently reduce the conversion of pro-IL-1β to mature IL-1β (66). Additionally, administration of CR might reduce depressive behavior by inhibiting microglial activity in the release of pro-inflammatory drugs, e.g., IL-1β and TNF-α (67). 

## Conclusion

Long-term infection with *T. gondii* caused affective disorders in the animals. Anxiety- and depressive-like behaviors were associated with OS condition due to the reduced levels of anti-oxidant enzymes (SOD and GSH) and increased levels of MDA, a ROS-induced lipid peroxidation factor. In addition, OS might be related to higher levels of proinflammatory mediators, including IL-1β, IL-6, IL-18, and TNF-α in the hippocampus of animals with *T. gondii *infection. Our findings revealed that curcumin alleviated anxiety- and depressive-like behaviors in *T. gondii*-infected animals by regulating OS biomarkers and modulating proinflammatory mediators. 

## Authors’ Contributions

ND and MZ contributed substantially to the conception and design of the study. FM, FBN, and AF performed the experiment, ND analyzed the data, FM, AF, and FBN drafted or provided critical revision of the article. MZ provided the final approval of the version to publish. All authors discussed the results and contributed to the final manuscript.

## Funding

This study was supported by Tehran University of Medical Sciences (1400-1-102-52864).

## Data Availability Statements

The datasets generated during and/or analyzed during the current study are available from the corresponding author upon reasonable request.

## Ethics Approval

 The research reported in this publication was approved by the Ethics Committee of Tehran University of Medical Sciences, Tehran, Iran (IR.TUMS.SPH.REC.1400.141).

## Conflicts of Interest

The authors declare that they have no competing interests.

## References

[1] Carruthers VBJAt (2002). Host cell invasion by the opportunistic pathogen Toxoplasma gondii.

[2] Verma R, Khanna P (2013). Development of Toxoplasma gondii vaccine: A global challenge. Hum Vaccin Immunother.

[3] Severance EG, Xiao J, Jones-Brando L, Sabunciyan S, Li Y, Pletnikov M (2016). Toxoplasma gondii—a gastrointestinal pathogen associated with human brain diseases. Int Rev Neurobiol.

[4] Saki J, Sabaghan M, Arjmand R, Teimoori A, Rashno M, Saki G (2020). Curcumin as an indirect methylation inhibitor modulates the effects of Toxoplasma gondii on genes involved in male fertility. Excli J.

[5] Postolache TT, Wadhawan A, Rujescu D, Hoisington AJ, Dagdag A, Baca-Garcia E (2021). Toxoplasma gondii, suicidal behavior, and intermediate phenotypes for suicidal behavior. Front Psychiatry.

[6] Nasirpour S, Kheirandish F, Fallahi S (2020). Depression and Toxoplasma gondii infection: Assess the possible relationship through a seromolecular case–control study. Arch Microbiol.

[7] Erickson LD, Brown BL, Gale SD, Hedges DW (2021). Association between Toxoplasma gondii seropositivity and serointensity and brain volume in adults: A cross-sectional study. PLoS One.

[8] Tedford E, McConkey G (2017). Neurophysiological changes induced by chronic Toxoplasma gondii infection. Pathogens.

[9] Xiao J, Yolken RH (2015). Strain hypothesis of Toxoplasma gondii infection on the outcome of human diseases. Acta Physiol (Oxf).

[10] Machado VS, Bottari NB, Baldissera MD, Rech VC, Ianiski FR, Signor C (2016). Diphenyl diselenide supplementation in infected mice by Toxoplasma gondii: Protective effect on behavior, neuromodulation and oxidative stress caused by disease. Exp Parasitol.

[11] Vilar-Pereira G, Silva AA, Pereira IR, Silva RR, Moreira OC, de Almeida LR (2012). Trypanosoma cruzi-induced depressive-like behavior is independent of meningoencephalitis but responsive to parasiticide and TNF-targeted therapeutic interventions. Brain Behav Immun.

[12] Vilar-Pereira G, Castaño Barrios L, Silva AAd, Martins Batista A, Resende Pereira I, Cruz Moreira O (2021). Memory impairment in chronic experimental Chagas disease: Benznidazole therapy reversed cognitive deficit in association with reduction of parasite load and oxidative stress in the nervous tissue. PloS One.

[13] Castaño Barrios L, Da Silva Pinheiro AP, Gibaldi D, Silva AA, Machado Rodrigues e Silva P, Roffê E (2021). Behavioral alterations in long-term Toxoplasma gondii infection of C57BL/6 mice are associated with neuroinflammation and disruption of the blood brain barrier. Plos one.

[14] Dunay IR, Gajurel K, Dhakal R, Liesenfeld O, Montoya JG (2018). Treatment of toxoplasmosis: Historical perspective, animal models, and current clinical practice. Clin Microbiol Rev.

[15] Remington JS ( 2006). Infectious diseases of the fetus and newborn infant.

[16] Petersen E, Schmidt DR (2003). Sulfadiazine and pyrimethamine in the postnatal treatment of congenital toxoplasmosis: what are the options?. Expert Rev Anti Infect Ther.

[17] Evangelista FF, Costa-Ferreira W, Mantelo FM, Beletini LF, de Souza AH, de Laet Sant’Ana P (2021). Rosuvastatin revert memory impairment and anxiogenic-like effect in mice infected with the chronic ME-49 strain of Toxoplasma gondii. PLoS One.

[18] Attari F, Zahmatkesh M, Aligholi H, Mehr SE, Sharifzadeh M, Gorji A (2015). Curcumin as a double-edged sword for stem cells: dose, time and cell type-specific responses to curcumin. Daru.

[19] Aggarwal BB, Sundaram C, Malani N, Ichikawa H (2007). Curcumin: The Indian solid gold. Adv Exp Med Biol.

[20] Azami SJ, Teimouri A, Keshavarz H, Amani A, Esmaeili F, Hasanpour H (2018). Curcumin nanoemulsion as a novel chemical for the treatment of acute and chronic toxoplasmosis in mice. Int J Nanomedicine.

[21] Ramaholimihaso T, Bouazzaoui F, Kaladjian A (2020). Curcumin in Depression: Potential Mechanisms of Action and Current Evidence—A Narrative Review. Front Psychiatry.

[22] Atia AF, Beshay EV, Fath-Allah SK, Sweed D, El-Refai SAJAP (2022). Recombinant mouse prolactin confers partial protection against Toxoplasma gondii infection in a pre-treated experimental murine model. Acta Parasitol.

[23] Ak T, Gülçin İJC-bi (2008). Antioxidant and radical scavenging properties of curcumin. Chem Biol Interact.

[24] Amiri S, Haj-Mirzaian A, Momeny M, Amini-Khoei H, Rahimi-Balaei M, Poursaman S (2017). Streptozotocin induced oxidative stress, innate immune system responses and behavioral abnormalities in male mice. Neuroscience.

[25] Wang D, An SC, Zhang X (2008). Prevention of chronic stress-induced depression-like behavior by inducible nitric oxide inhibitor. Neuroscience Lett.

[26] Porsolt RD, Bertin A, Jalfre M (1977). Behavioral despair in mice: a primary screening test for antidepressants. Arch Int Pharmacodyn Ther.

[27] Yankelevitch-Yahav R, Franko M, Huly A, Doron R (2015). The forced swim test as a model of depressive-like behavior. J Vis Exp.

[28] Bhadra R, Cobb DA, Weiss LM, Khan IA (2013). Psychiatric disorders in toxoplasma seropositive patients--the CD8 connection. Schizophr Bull.

[29] Bay-Richter C, Petersen E, Liebenberg N, Elfving B, Wegener G (2019). Latent toxoplasmosis aggravates anxiety- and depressive-like behaviour and suggest a role of gene-environment interactions in the behavioural response to the parasite. Behav Brain Res.

[30] Xiao J (2020). Toxoplasma-induced Behavioral Changes: An Aspecific Consequence of Neuroinflammation. Trends Parasitol.

[31] Burgdorf KS, Trabjerg BB, Pedersen MG, Nissen J, Banasik K, Pedersen OB (2019). Large-scale study of Toxoplasma and Cytomegalovirus shows an association between infection and serious psychiatric disorders. Brain Behav Immun.

[32] Wang T, Tang Z-h, Li J-f, Li X-n, Wang X, Zhao Z-j (2013). A potential association between Toxoplasma gondii infection and schizophrenia in mouse models. Exp Parasitol.

[33] Juszczyk G, Mikulska J, Kasperek K, Pietrzak D, Mrozek W, Herbet M (2021). Chronic stress and oxidative stress as common factors of the pathogenesis of depression and Alzheimer’s disease: The role of antioxidants in prevention and treatment. Antioxidants.

[34] Bouayed J, Rammal H, Soulimani R (2009). Oxidative stress and anxiety: relationship and cellular pathways. Oxid Med Cell Longev.

[35] Zhou R, Tardivel A, Thorens B, Choi I, Tschopp J (2010). Thioredoxin-interacting protein links oxidative stress to inflammasome activation. Nat Immunol.

[36] Anderson G, Berk M, Dodd S, Bechter K, Altamura AC, Dell’osso B (2013). Immuno-inflammatory, oxidative and nitrosative stress, and neuroprogressive pathways in the etiology, course and treatment of schizophrenia. Prog Neuropsychopharmacol Biol Psychiatry.

[37] Giridharan VV, Sayana P, Pinjari OF, Ahmad N, da Rosa MI, Quevedo J (2020). Postmortem evidence of brain inflammatory markers in bipolar disorder: a systematic review. Mol Psychiatry.

[38] Mokhtari T, Tu Y, Hu L (2019). Involvement of the hippocampus in chronic pain and depression. Brain Sci Adv.

[39] Troubat R, Barone P, Leman S, Desmidt T, Cressant A, Atanasova B (2021). Neuroinflammation and depression: A review. Eur J Neurosci.

[40] Crupi R, Cambiaghi M, Spatz L, Hen R, Thorn M, Friedman E (2010). Reduced adult neurogenesis and altered emotional behaviors in autoimmune-prone B-cell activating factor transgenic mice. Biol Psychiatry.

[41] Greenhalgh AD, David S, Bennett FC (2020). Immune cell regulation of glia during CNS injury and disease. Nat Rev Neurosci.

[42] Xu S, Lu J, Shao A, Zhang JH, Zhang J (2020). Glial cells: role of the immune response in ischemic stroke. Front Immunol.

[43] Johnson HJ, Koshy AA (2020). Latent toxoplasmosis effects on rodents and humans: how much is real and how much is media hype?. MBio.

[44] Li Y, Severance EG, Viscidi RP, Yolken RH, Xiao J (2019). Persistent Toxoplasma infection of the brain induced neurodegeneration associated with activation of complement and microglia. Infect Immun.

[45] Boillat M, Hammoudi P-M, Dogga SK, Pagès S, Goubran M, Rodriguez I (2020). Neuroinflammation-associated aspecific manipulation of mouse predator fear by Toxoplasma gondii. Cell Rep.

[46] O’Callaghan JP, Miller DB (2019). Neuroinflammation disorders exacerbated by environmental stressors. Metabolism.

[47] Hsu PC, Groer M, Beckie T (2014). New findings: depression, suicide, and Toxoplasma gondii infection. J Am Assoc Nurse Pract.

[48] Mahmoudvand H, Ziaali N, Ghazvini H, Shojaee S, Keshavarz H, Esmaeilpour K (2016). Toxoplasma gondii infection promotes neuroinflammation through cytokine networks and induced hyperalgesia in BALB/c mice. Inflammation.

[49] Song C, Wang H (2011). Cytokines mediated inflammation and decreased neurogenesis in animal models of depression. Prog Neuropsychopharmacol Biol Psychiatry.

[50] Dowlati Y, Herrmann N, Swardfager W, Liu H, Sham L, Reim EK (2010). A meta-analysis of cytokines in major depression. Biol Psychiatry.

[51] Rossi S, Sacchetti L, Napolitano F, De Chiara V, Motta C, Studer V (2012). Interleukin-1β causes anxiety by interacting with the endocannabinoid system. J Neurosci.

[52] Hannestad J, DellaGioia N, Bloch M (2011). The effect of antidepressant medication treatment on serum levels of inflammatory cytokines: A meta-analysis. Neuropsychopharmacology.

[53] Crupi R, Cambiaghi M, Deckelbaum R, Hansen I, Mindes J, Spina E (2012). n− 3 fatty acids prevent impairment of neurogenesis and synaptic plasticity in B-cell activating factor (BAFF) transgenic mice. Prev Med.

[54] Aggarwal BB, Sung B (2009). Pharmacological basis for the role of curcumin in chronic diseases: an age-old spice with modern targets. Trends Pharmacol Sci.

[55] Magalhães LG, Machado CB, Morais ER, de Carvalho Moreira ÉB, Soares CS, da Silva SH (2009). In vitro schistosomicidal activity of curcumin against Schistosoma mansoni adult worms. Parasitol Res.

[56] Chen Y-Q, Xu Q-M, Li X-R, Yang S-L, Zhu-Ge H-X (2012). In vitro evaluation of schistosomicidal potential of curcumin against Schistosoma japonicum. J Asian Nat Prod Res.

[57] Pérez-Arriaga L, Mendoza-Magana M, Cortés-Zárate R, Corona-Rivera A, Bobadilla-Morales L, Troyo-Sanromán R (2006). Cytotoxic effect of curcumin on Giardia lamblia trophozoites. Acta Trop.

[58] Saleheen D, Ali SA, Ashfaq K, Siddiqui AA, Agha A, Yasinzai MM (2002). Latent activity of curcumin against leishmaniasis in vitro. Biol Pharm Bull.

[59] Chakrabarti R, Rawat PS, Cooke BM, Coppel RL, Patankar S (2013). Cellular effects of curcumin on Plasmodium falciparum include disruption of microtubules. PLoS One.

[60] Nagajyothi F, Zhao D, Weiss LM, Tanowitz HB (2012). Curcumin treatment provides protection against Trypanosoma cruzi infection. Parasitol Res.

[61] Goo Y-K, Yamagishi J, Ueno A, Terkawi MA, Aboge GO, Kwak D (2015). Characterization of Toxoplasma gondii glyoxalase 1 and evaluation of inhibitory effects of curcumin on the enzyme and parasite cultures. Parasit Vectors.

[62] Mokhtari T (2022). Targeting autophagy and neuroinflammation pathways with plant-derived natural compounds as potential antidepressant agents. Phytother Res.

[63] Talei S, Mokhtari T, Asadi I, Arab L, Hassanzadeh N, Hosseinjani E (2021). Flaxseed oil (Linum Usitatissimum) attenuates restraint stress-induced depressive-like behavior: Upregulation of neurotrophic factors in CA1 region of hippocampus. J Contemp Med Sci.

[64] Dubey AK, Goyal S, Goswami A, Gupta PK, Gupta A (2021). Evaluation of the antidepressant potential of curcumin extract in mice. CNS Neurol Disord Drug Targets..

[65] Yin H, Guo Q, Li X, Tang T, Li C, Wang H (2018). Curcumin suppresses IL-1β secretion and prevents inflammation through inhibition of the NLRP3 inflammasome. J Immunol.

[66] Zhang W-y, Guo Y-j, Han W-x, Yang M-q, Wen L-p, Wang K-y (2019). Curcumin relieves depressive-like behaviors via inhibition of the NLRP3 inflammasome and kynurenine pathway in rats suffering from chronic unpredictable mild stress. Int Immunopharmacol.

[67] Wang Z, Zhang Q, Yuan L, Wang S, Liu L, Yang X (2014). The effects of curcumin on depressive-like behavior in mice after lipopolysaccharide administration. Behav Brain Res.

